# The antigen processing-associated transporter gene polymorphism: Role on gene and protein expression in HPV-infected pre-cancerous cervical lesion

**DOI:** 10.3389/fcimb.2022.979800

**Published:** 2022-12-21

**Authors:** Fernanda Silva Medeiros, Mauro César da Silva, Neila Caroline Henrique da Silva, Thailany Thays Gomes, Renan Garcia Gomes, Larissa Albuquerque Paiva, Fabiana Oliveira dos Santos Gomes, Christina Alves Peixoto, Maria Carolina Valença Rygaard, Stefan Welkovic, Maria Luiza Bezerra Menezes, Eduardo Antônio Donadi, Norma Lucena-Silva

**Affiliations:** ^1^ Laboratory of Immunogenetics, Department of Immunology, Aggeu Magalhães Institute, Oswaldo Cruz Foundation, Recife, Brazil; ^2^ Laboratory of Molecular Biology, Institute of Integral Medicine Professor Fernando Figueira (IMIP) Hospital, Pediatric Oncology Service, Recife, Brazil; ^3^ Getúlio Vargas Hospital, Pernambuco Health Department, Recife, Brazil; ^4^ Laboratory of Ultrastructure, Department of Entomology, Aggeu Magalhães Institute, Oswaldo Cruz Foundation, Recife, Brazil; ^5^ Integrated Health Center Amaury de Medeiros (CISAM), University of Pernambuco, Recife, Brazil; ^6^ Clinical Immunology Division, Department of Medicine, School of Medicine of Ribeirão Preto, University of São Paulo (USP), Ribeirão Preto, Brazil

**Keywords:** TAP1, TAP2, HPV, polymorphism, cervical lesion

## Abstract

Human papillomavirus (HPV) is the major pathogen for cervical lesions. The evasion mechanism of the immune response and persistence of HPV infection can be influenced by polymorphisms (SNPs) in genes associated with transporter associated with antigen processing (TAP), which may change the peptide binding affinity or the TAP expression impacting the efficiency of peptide transport in the secretory pathway, and the presentation of peptides to cytotoxic T lymphocytes. This study aimed to evaluate the role of the *TAP1* and *TAP2* polymorphisms, *TAP1*, and *TAP2* genes expressions, and protein levels in cervical cells presenting different degrees of pre-cancerous lesions in 296 immunocompetent women infected or not by HPV. *TAP* SNPs were genotyped by Sanger sequencing, and gene expression by real-time PCR. Aneuploidy was determined by DNA index using flow cytometry. TAP-1 and TAP-2 tissue expressions were evaluated by immunohistochemistry. The Asp697Gly SNP of *TAP1* presented a risk for cellular aneuploidy (P=0.0244). HPV+ women had higher TAP-2 mRNA (P=0.0212) and protein (P<0.0001) levels. The TAP2D and TAP2E haplotypes were associated with the risk for aneuploidy and pre-cancerous lesions. In conclusion, nucleotide variability at the peptide binding region of peptide transporter genes, particularly of the *TAP2* gene, may influence the HPV-peptide transportation from the cytosol to the endoplasmic reticulum, increasing the susceptibility to the development of high-grade cervical lesions.

## 1 Introduction

The immunological recognition of cancerous and virus-infected cells requires the degradation of endogenous protein into peptides *via* the ubiquitin/proteasome and other proteolytic pathways, which are translocated from the cytosol into the lumen of the endoplasmic reticulum (ER) by the transporter-associated with antigen processing (TAP) (Lehnert and Tampé, 2017). In the ER, the peptide binds to the classical HLA class I (HLA-I) molecules to be further transported to the membrane surface for recognition by CD8+ cytotoxic T cells (CTLs) and NK cells (Thomas and Tampé, 2021).

The presence of single nucleotide polymorphisms (SNPs) in *TAP* genes may influence the selection of peptides bound to HLA-I molecules, modulating the diversity of peptide transport from cytosol to ER (Colonna et al., 1992; Lehnert and Tampé, 2017). Most SNPs in *TAP* genes (*TAP1* and *TAP2*) are close to the peptide-binding site, and some are in the protein transmembrane domain (Praest et al., 2018). These coding region SNPs at the *TAP1* and *TAP2* genes have been associated with rheumatoid arthritis (Zhang et al., 2002), systemic lupus erythematosus (Correa et al., 2003), esophageal cancer (Zou et al., 2015; Guo et al., 2016), multiple myeloma and chronic lymphocytic leukemia (Ozbas-Gerceker et al., 2013), and tuberculosis (Sunder et al., 2011; Thu et al., 2016). Additionally, *TAP* gene SNPs have been associated with cervical precursor lesions of cervical cancer (CC) (Einstein et al., 2009; Natter et al., 2013).

Human papillomavirus (HPV) is a sexually transmitted infection, accounting for 99.7% of CC cases (Walboomers et al., 1999). In Brazil, CC is the third most common cancer among women aged 15 to 44 (Bray et al., 2018). The host’s immune system clears most cervical HPV infections within 2 to 3 years; however, in many cases, the infection can persist longer, leading to low-grade (LSIL) and high-grade lesions (HSIL). The histological lesions are classified as cervical intraepithelial neoplasia (CIN) 1, 2, or 3, which can progress to invasive cancer (Oyervides-Muñoz et al., 2018). Primary cancer transforming factors mediated by high-risk oncogenic HPV (HrHPV), particularly E6 and E7 oncoproteins, interact with the host cells to manipulate cellular processes and promote cell transformation (Pal and Kundu, 2020). Little attention has been devoted to the relationship between HPV infection and CINs associated with the *TAP* gene variability.

To understand the contribution of TAP on HPV-induced cervical lesions, we analyzed five SNPs at the coding regions of *TAP* genes and evaluated the *TAP* genes expression and protein levels in cervical samples obtained from women infected or not by HPV.

## 2 Material and methods

### 2.1 Study population

We enrolled consecutively 296 women (38.6 ± 8.9 years) at the Cervical Pathology outpatient clinic of the Instituto de Medicina Integral Professor Fernando Figueira (IMIP) and Colposcopy Service at the Centro Integrado de Saúde Amaury de Medeiros (CISAM) in Recife (Northeast Brazil), between April 2016 and October 2018. All patients were from the metropolitan region of the Recife capital and were 18 years or older. They were recruited before undergoing colposcopy and oncotic cytology examination, offered free of charge to every woman in the public health service by the National Cervical Cancer Surveillance Program of the Brazilian Health Ministry. Thus, the research team did not have access to information about a previous diagnosis of cervical lesion or HPV infection at the time of recruiting the volunteers for the study. All participants signed the informed consent form. The Ethics Committee of the Aggeu Magalhães Institute approved this study under the protocol CAAE:51111115.9.0000.5190. Clinical and laboratory data of the patients were obtained from the hospital records (**Table 1**).

**Table 1:** Clinical characteristics of women treated at two Cervical Pathology Outpatient Clinics in Recife, Northeast Brazil.

Patients’ characteristics	Total	
	N = 296	(%)
Age, years		
Mean ( ± SD)	38.6 ( ± 8.9)	
HPV infection	296	
Yes	107	(36.1)
No	189	(63.9)
Cytological alterations		
No atypia	99	(39.4)
ASC-US and ASC-H	24	(9.6)
LSIL	53	(21.1)
HSIL	75	(29.9)
Data missing	45	
Histological alterations		
No indication for biopsy#	130	(51.6)
Benign lesion	36	(14.3)
CIN 1	21	(8.3)
CIN 2	31	(12.3)
CIN 3	34	(13.5)
Data missing	44	
Cellular ploidy		
Aneuploidy	22	(18.8)
Diploidy	95	(81.2)
Data missing	179	

Number of individuals (N); low-grade squamous intraepithelial lesion (LSIL); high-grade squamous intraepithelial lesion (HSIL); cervical intraepithelial neoplasia (CIN); atypical squamous cells of undifferentiated (ASC-US), atypical squamous cells not excluding high-grade squamous intraepithelial lesion (ASC-H). #According to the Brazilian Ministry of Health screening policy for cervical cancer, there is no indication for colposcopy for these patients due to the absence of changes in the cytological examination (da Silva et al., 2021).

### 2.2 Sample processing and characterization

After answering a clinical questionnaire, the patient’s peripheral blood was collected to study the association of inherited gene variants with precancerous cervical disease. Then, during the colposcopy examination, we collected a sample of exfoliative cervical cells for molecular diagnosis of HPV infection, evaluation of the DNA ploidy, and gene expression studies. Therefore, they were linked to the cytological and histological examination of the day they were collected.

In the presence of atypia, a cervical biopsy was performed for further histopathological and immunohistochemical analysis, justifying why a small number of women were evaluated for TAP protein expression in the cervical lesions. The colposcopy results were classified according to the nomenclature of the International Federation for Cervical Pathology and Colposcopy, IFCPC (Bornstein et al., 2012). The absence of CIN meant that the cervix showed no alterations in the colposcopy or exhibited no lesion in the biopsy (n=130). Biopsies were classified as benign lesions (n=36), low-grade lesions (CIN 1 = 21), and high-grade lesions (CIN 2 = 31 and CIN 3 = 34). We wrote down the results of cytology and histology from the medical record, and 44 histology results were missing.

Genomic DNA was extracted from cervical samples according to the methodology reported by Medeiros et al. (2022) (Medeiros et al., 2022). PCR amplification of the viral *L1* gene confirmed the HPV infection, and the sequence of the *L1* fragment defined the HPV type. Of the 296 women evaluated, 189 were negative for HPV infection (HPV-), and 107 were positive for HPV (HPV+). Of these, 11 (10.3%) were infected with low-risk HPV (HPV 6, 11, 61, 72, 81, and 83), and 96 (89.7%) with HrHPV (HPV 16, 18, 31, 33, 51, 52, 53, 58, 59, and 66).

The DNA ploidy of the exfoliative cervical cells was determined as previously reported (da Silva et al., 2021). A DNA index greater than 1.16 (hyperploid) or less than 1.00 (hypoploid) with more than 10% of the cell population analyzed in the area corresponding to G0-G1 of the cell cycle defined aneuploidy (Martins et al., 2014).

### 2.3 Polymorphisms at the coding regions of the *TAP* and *TAP* genes

Peripheral blood mononuclear cells (PBMC) were isolated by gradient density using Ficoll-Paque™ Plus reagent (GE Healthcare, Little Chalfont, UK) and submitted to *g*DNA extraction using the DNAzol reagent (Invitrogen, Carlsbad, CA). Conventional PCR used specific primers designed according to the target region at *TAP1* (Genbank ID: 6890) (Fang et al., 2017) and *TAP2* (Genbank ID: 6891) genes. Primers and reaction conditions are shown in **Table S1**. PCR fragment sequencing followed the Big Dye Terminator v 3.1 protocol, performed using the ABI 3500 sequencer (Applied Biosystems, Waltham, MA). Sequence alignment against the *TAP1* and *TAP2* genes used the SeqMan v.7.0 program (DNASTAR, Madison, WI) for the genotype identification. We evaluated the SNP rs1135216 in exon 10 at *TAP1* and the SNPs rs2228396 in exon 10, and rs4148876 and rs241447 in exon 12 at *TAP2* that are in the peptide binding site; and the SNP rs1800454 in exon 6 at *TAP2* related to the peptide transport.

### 2.4 Gene expression of *TAP1* and *TAP2*

The total RNA extraction of exfoliative cervical cells was performed using the TRIzol^®^ reagent and cDNA synthesis using the M-MLV RT reverse transcriptase enzyme (Invitrogen) following the manufacturer’s protocol. Specific primers designed to amplify the mRNA of the *TAP1* (ID: NM_000593.5) and the mRNA of the *TAP2* (ID: NM_000544) genes used the Oligo IDT software. The *TAP1* and *TAP2* expression was quantified in duplicates by qPCR in the QuantStudio™ 5 instrument (Applied Biosystems) following the reaction parameters described in **Table S1**. The reference used was the level of the constitutive glyceraldehyde-3-phosphate dehydrogenase (*GAPDH*) gene expression (Marques et al., 2011). Primers and reaction conditions are shown in **Table S1**. Thresholds were established for each target separately, and only the amplification graph duplicates that differed between the quantification cycles (Cq) less than 0.5 were analyzed. Duplicates presenting only a single peak in the melting curve reached the criterium of specificity for analyses. The *TAP1* and *TAP2* relative expression was obtained through the mean Cq values of duplicates of each target and normalized by the Cq of the reference gene (*GAPDH*). The formula for each sample was: ΔCq = target average Cq, reference average Cq. The relative changes in gene expression used the Fold-change = 2-ΔCq.

### 2.5 Histology and immunohistochemistry analyses

Biopsies of the cervical lesions of 40 women were fixed in 10% buffered formalin and embedded in paraffin. Slides of 4μm tissue sections were stained with hematoxylin and eosin (H&E), assembled with Entellan^®^ (MERCK, Burlington, MA), and visualized with 400x magnification in an inverted microscope (Zeiss, Göttingen, Germany) equipped with a camera and a 4.7.4 Image Analysis Program (AxionCam MRm, Zeiss). Immunohistochemistry (IHC) reaction used the Dako EnVision™ FLEX+ Kit (Agilent-Dako, Code: K8002, Dako Laboratories, Carpinteria, CA) following the manufacturer’s instructions. After deparaffinization and hydration, the slides were subjected to heat-induced antigen retrieval with Tris/EDTA Buffer pH 9 for 30 minutes. After the inactivation of endogenous peroxidase by the peroxidase-blocking reagent, the slides were incubated in a humid chamber, at room temperature, with the following primary antibodies: rabbit polyclonal anti-TAP-1 (ab83817; Abcam, Cambridge, UK) and rabbit polyclonal anti-TAP-2 (ab180611; Abcam). Phosphate-buffered saline (PBS) replaced the primary antibody as a negative control. The sections were incubated for 20 minutes with the EnVision™ FLEX/HRP, and the reaction was visualized with the chromogen 3,3’-diaminobenzidine tetrahydrochloride (DAB) solution. The cells were counterstained with Harris’ hematoxylin. Two experienced pathologists blind-evaluated the labeling for TAP-1 and TAP-2. Brown staining detected in the cytoplasm of cells was considered positive for TAP-1 or TAP-2. Three photos of each slide were captured per patient. The protein levels were estimated by the Gimp 2.10 software (GNU Image Manipulation Program, UNIX platforms, www.gimp.org), considering the intensity of pixels in the stained area of the image.

### 2.6 Statistical analysis

The description of the categorical variables used absolute number and frequency (%), and the comparisons were performed using Fisher’s exact or the Chi-square tests. Comparison of continuous variables between two groups used the student-T or the Mann-Whitney-U tests. In contrast, comparisons of three or more groups were analyzed using the ANOVA or Kruskal-Wallis tests. The decision to use parametric or non-parametric tests was based on the distribution of the variables using the Kolmogorov-Smirnov normality test. The statistical analyses used the GraphPad Prism program (version 5.0) for Windows (GraphPad Software, San Diego, CA). The association between genetic frequencies with susceptibility to the disease was determined by calculating the Odds Ratio (OR) using Fisher’s exact test, considering P<0.05 as significant. The Hardy-Weinberg equilibrium (HWE) expectations were assessed using specific calculator software (https://wpcalc.com/en/equilibrium-hardy-weinberg/). The linkage disequilibrium (LD) was evaluated by the r^2^ statistic between different polymorphisms using the Haploview software (https://www.broadinstitute.org/haploview/haploview).

## 3 Results

### 3.1 Association of the *TAP* Asp697Gly variant with gene and protein expression in cervical lesions

The p.Asp697Gly (rs1135216) allele and genotype frequencies were not associated with the cytological and histological lesions or with the presence of HPV infection in 290 women (P>0.05, data not shown), and the genotypes distribution fitted the HWE equilibrium expectations (χ^2 =^ 0.0399). Noteworthy, carriers of the rare GG genotype were at increased risk for cervical cell aneuploidy (OR=6.7, 95%CI =1.4-32.4, P=0.0244) (**Table 2**).

**Table 2:** Frequency of the alleles and genotypes of the p.Asp697Gly SNP in the *TAP1* gene, stratified according to cell ploidy.

TAP1SNP rs1135216	^a^ Patients		Cell ploidy					P	OR (CI-95%)
			Aneuploidy			Diploidy			
Genotypes (A>G)	N = 290	(%)	N = 22	(%)		N = 93	(%)	
** AA**	194	(66.9)	16	(72.7)		58	(62.4)	Reference	
** AG**	87	(30.0)	2	(9.1)		32	(34.4)	0.052	4.4 (1.0-20.1)
** GG**	9	(3.1)	4	(18.2)		3	(3.2)	0.059	0.2 (0.0-0.8)
Dominant model									
** AA**	194	(66.9)	16	(72.7)		58	(62.4)	0.4611	0.6 (0.2-1.7)
** (AG+GG)**	96	(33.1)	6	(27.3)		35	(37.6)	Reference	
Recessive model									
** GG**	9	(3.1)	4	(18.2)		3	(3.2)	**0.0244**	**6.7 (1.4-32.4)**
** (AA+AG)**	281	(96.9)	18	(81.8)		90	(96.8)	Reference	
Alleles	N = 580	%	N = 44	%		N = 186	%		
** A**	475	(81.9)	34	(77.3)		148	(79.6)	Reference	
** G**	105	(18.1)	10	(22.7)		38	(20.4)	0.8366	0.8 (0.4-1.9)

a Number of patients studied. Confidence Interval (CI). Odds Ratio (OR). Single Nucleotide Polymorphism (SNP). The P value was calculated by Fisher’s exact test and P<0.05 was considered statistically significant. Results highlighted in bold are statistically significant.

**Figure 1:**
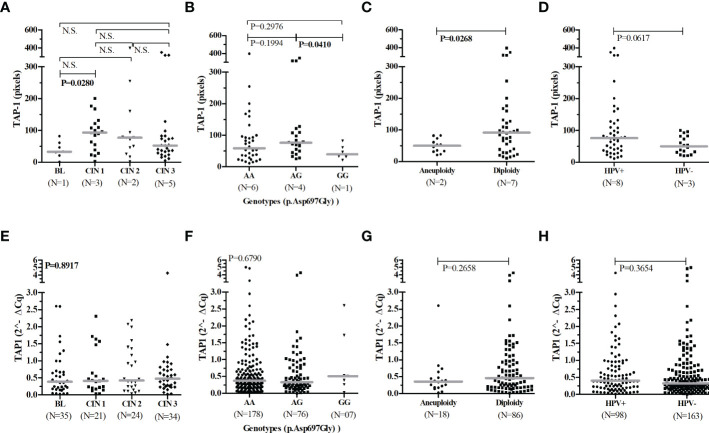
TAP-1 protein levels and *TAP1* gene expression stratified according to the severity of the cervical lesions, Asp697Gly SNP genotypes, chromosomal number, and HPV infection. Number of patients analyzed (N). The Mann-Whitney and Kruskal-Wallis tests were used to estimate the differences between the expressions of TAP-1 protein and *TAP1* mRNA in the evaluated groups. TAP-1 protein expression **(A–D)** in cervical biopsies, and *TAP1* gene expression **(E–H)** in cervical exfoliative samples.

The *TAP1* mRNA expression in exfoliative cervical cells of 261 women showed no significant difference in the median levels of *TAP1* transcripts for the following groups of comparison: aneuploidy *vs*. diploidy (N=104; P=0.2658); HPV+ *vs*. HPV- (N=261; P=0.3654); cytological (N=224; P=0.1813) or histological (N=228; P=0.6656) alterations; and *TAP1* p.Asp697Gly genotypes (N=261; P=0.6790) (**Figures 1** **E–H**).

Overall, the TAP-1 protein was higher in CIN 1 lesions compared to BL (P=0.0280) (**Figure 1** **A**). Carriers of the GG genotype exhibited low levels of TAP-1 when compared to carriers of the AG genotype (P=0.0410). However, the number of biopsies studied of women showing the genotype GG was negligible (**Figure 1** **B**). Low TAP-1 protein levels were associated with aneuploidy (P=0.0268) (**Figure 1** **C**). HPV+ samples exhibited increased levels of TAP-1 compared to HPV- samples; however, significance was not reached (P=0.0617) (**Figure 1** **D**).

### 3.2 Association of the *TAP2* variants with gene and protein expression in cervical lesions

The *TAP2* alleles and genotype frequencies are shown in **Table 3**. The frequency of the genotypes of the SNP Val379Ile (rs1800454) was in HWE (χ^2 =^ 1.7), but the genotypes of the SNPs Ala565Thr (rs2228396) (χ^2 =^ 57.2), the Arg651Cys (rs4148876) (χ^2 =^ 83.5), and the Thr665Ala (rs241447) (χ^2 =^ 268.9) did not fit the expected proportions of HWE. In addition, the SNPs rs241447 (Thr665Ala) and rs2228396 (Ala565Thr) are in linkage disequilibrium (D’=100).

**Table 3:** The *TAP2* genetic variants alleles and genotypes frequencies according to cell ploidy.

TAP2 gene		^a^ Patients		Cell ploidy						P	OR (CI-95%)
				Aneuploidy			Diploidy				
rs1800454 (p.Val379Ile)											
**Genotypes (G>A)**		**N = 295**	**(%)**	**N = 22**		**(%)**	**N = 95**		**(%)**		
**GG**		223	(75.6)	11		(50.0)	78		(82.1)	Reference	
**GA**		64	(21.7)	8		(36.4)	16		(16.8)	**0.0274**	**3.5 (1.2-10.2)**
**AA**		8	(2.7)	3		(13.6)	1		(1.1)	**0.0102**	**21.3 (2.0-223.1)**
Dominant model											
**GG**		223	(75.6)	11		(50.0)	78		(82.1)	Reference	
**(GA+AA)**		72	(24.4)	11		(50.0)	17		(17.9)	**0.0040**	**4.5 (1.7-12.3)**
Recessive model											
**(GG+GA)**		287	(97.3)	19		(86.4)	94		(98.9)	Reference	
**AA**		8	(2.7)	3		(13.6)	1		(1.1)	**0.0207**	**14.8 (1.5-150.5)**
**Alleles**		N = 590	(%)	N = 44		(%)	N = 190		(%)		
**G**		510	(86.4)	30		(68.2)	172		(90.5)	Reference	
**A**		80	(13.6)	14		(31.8)	18		(9.5)	**0.0004**	**4.4 (2.0-9.9)**
rs2228396 (p.Ala565Thr)											
**Genotypes (G>A)**		**N = 289**	**(%)**	**N = 21**		**(%)**	**N = 95**		**(%)**		
**GG**		234	(81.0)	13		(61.9)	80		(84.2)	Reference	
**GA**		35	(12.1)	4		(19.0)	11		(11.6)	0.2499	0.4 (0.1-1.6)
**AA**		20	(6.9)	4		(19.0)	4		(4.2)	**0.0259**	**6.1 (1.3-27.7)**
Dominant model											
**GG**		234	(81.0)	13		(61.9)	80		(84.2)	Reference	
**(GA+AA)**		55	(19.0)	8		(38.1)	15		(15.8)	**0.0321**	**3.3 (1.2-9.3)**
Recessive model											
**(GG+GA)**		269	(93.1)	17		(81.0)	91		(95.8)	Reference	
**AA**		20	(6.9)	4		(19.0)	4		(4.2)	**0.0348**	**5.3 (1.21-23.5)**
**Alleles**		N = 578	(%)	N = 42		(%)	N = 190		(%)		
**G**		503	(87.0)	30		(71.4)	171		(90.0)	Reference	
**A**		75	(13.0)	12		(28.6)	19		(10.0)	**0.0042**	**3.6 (1.6-8.2)**
(conclusion)											
rs4148876 (p.Arg651Cys)											
**Genotypes (C>T)**	**N = 284**		**(%)**	**N = 21**	**(%)**		**N = 93**	**(%)**			
**CC**	273		(96.1)	20	(95.2)		90	96.8)		Reference	
**CT**	8		(2.8)	1	(4.8)		1	(1.1)		0.3412	0.2 (0.0-3.7)
**TT**	3		(1.1)	0	(0.0)		2	(2.2)		1.0000	1.1 (0.0-24.5)
Dominant model											
**CC**	273		(96.1)	20	(95.2)		90	(96.8)		Reference	
**(CT+TT)**	11		(3.9)	1	(4.8)		3	(3.2)		0.5624	0.6 (0.0-6.7)
Recessive model											
**(CC+CT)**	281		(98.9)	21	(95.2)		91	(97.8)		Reference	
**TT**	3		(1.1)	0	(4.8)		2	(2.2)		1.0000	1.2 (0.0-25.3)
**Alleles**											
**C**	554		(97.5)	41	(97.6)		181	(97.3)		Reference	
**T**	14		(2.5)	1	(2.4)		5	(2.7)		1.0000	1.1 (0.1-9.9)
rs241447 (p.Thr665Ala)											
**Genotypes (A>G)**	**N = 284**		**(%)**	**N = 21**	**(%)**		**N = 93**	**(%)**			
**AA**	262		(92.3)	20	(95.2)		83	(89.2)		Reference	
**AG**	1		(0.4)	0	(0.0)		1	(1.1)		1.0000	0.7 (0.0-18.7)
**GG**	21		(7.4)	1	(4.8)		9	(9.7)		0.6852	2.1 (0.2-18.1)
Dominant model											
**AA**	262		(92.3)	20	95.2		83	89.2		Reference	
**(AG+GG)**	22		(7.7)	1	4.8		10	10.8		0.6861	2.4 (0.3-19.9)
Recessive model											
**(AA+AG)**	263		(92.6)	20	95.2		84	90.3		Reference	
**GG**	21		(7.4)	1	4.8		9	9.7		0.6858	2.1 (0.2-17.9)
Alleles											
**A**	525		(92.4)	40	95.2		167	89.8		Reference	
**G**	43		(7.6)	2	4.8		19	10.2		0.3812	2.3 (0.5-10.1)

a Number of patients studied. Confidence Interval (CI). Odds Ratio (OR). Single Nucleotide Polymorphism (SNP). The P value was calculated by Fisher’s exact test and P<0.05 was considered significant. Results highlighted in bold are statistically significant.

The *TAP2* gene expression, stratified according to the alleles of each of the four studied *TAP2* SNP (Val379Ile, Ala565Thr, Arg651Cys, or Thr665Ala), showed no significant results (**Figure S1**). However, the presence of the rare p.Val379Ile allele A in a dominant model (GA+AA) increased the TAP-2 protein levels according to the lesion severity. The p.Ala565Thr allele A also increased the protein level in the cervical lesion, except in the CIN 3 (data not shown).

The presence of the rare allele A in the p.Val379Ile and p.Ala565Thr SNPs increased the risk of aneuploidy in the allelic (OR=4.4, 95%CI=2.0-9.9, P=0.0004; and OR=3.6, 95%CI=1.6-8.2, P=0.0042), dominant (OR=4.5, 95%CI=1.7-12.3, P=0.0040; and OR=3.3, 95%CI=1.2-9.3, P=0.0321), and recessive (OR=14.8, 95%Cl=1.5-150.5, P=0.0207; and OR=5.3, 95%CI=1.2-23.5, P=0.0348) models (**Table 3**).

**Figure 2:**
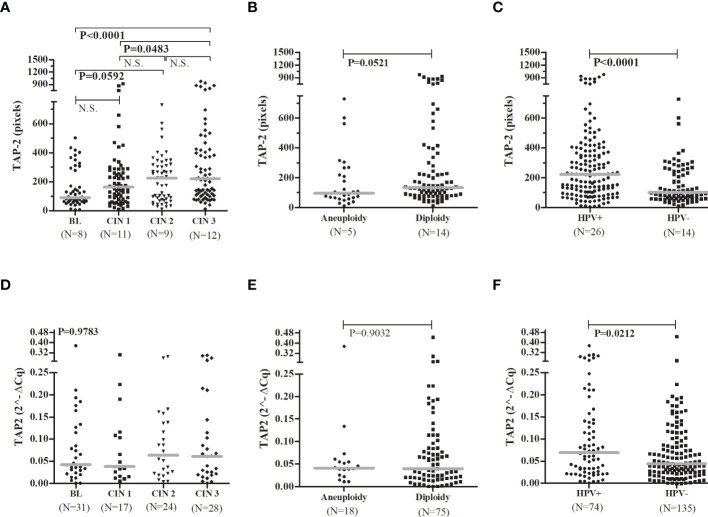
TAP-2 protein levels and *TAP2* gene expression, stratified according to the severity of the cervical lesions, the chromosomal number, and the HPV infection.Legend: Number of patients analyzed (N). Note: The Mann-Whitney or Kruskal-Wallis tests were used to estimate the differences between the expressions of TAP-2 protein and *TAP2* mRNA in the evaluated groups. TAP-2 protein expression **(A–C)** in cervical biopsy samples and *TAP2* gene expression **(D–F)** in cervical exfoliative samples.

In addition, the presence of the rare allele G in the p.Thr665Ala SNP increased the chance of presenting HSIL compared to other atypia (ASC-US, ASC-H, and LSIL) (OR= 3.7, 95%CI =1.5-9.6, P= 0.0038), and increased the risk of CIN (OR= 3.6, 95%CI = 1.2-10.3, P= 0.0119) (data not shown). Interestingly in women with HSIL, the rare homozygous p.Thr665Ala GG genotype was associated with a significant decrease in *TAP2* mRNA levels in exfoliative cervical cells. However, overall *TAP2* mRNA levels did not differ accordingly to the severity of histological lesions (BL, CIN 1, CIN 2, or CIN 3, N=100 patients; P=0.9783) (**Figure 2D**) or cell ploidy (N=93 patients; P=0.9032) (**Figure 2E**). The *TAP2* mRNA in exfoliative cervical cells showed higher expression of *TAP2* transcripts in HPV+ compared to HPV- samples (N=209 patients; P=0.0212) (**Figure 2F**).

**Figure 3:**
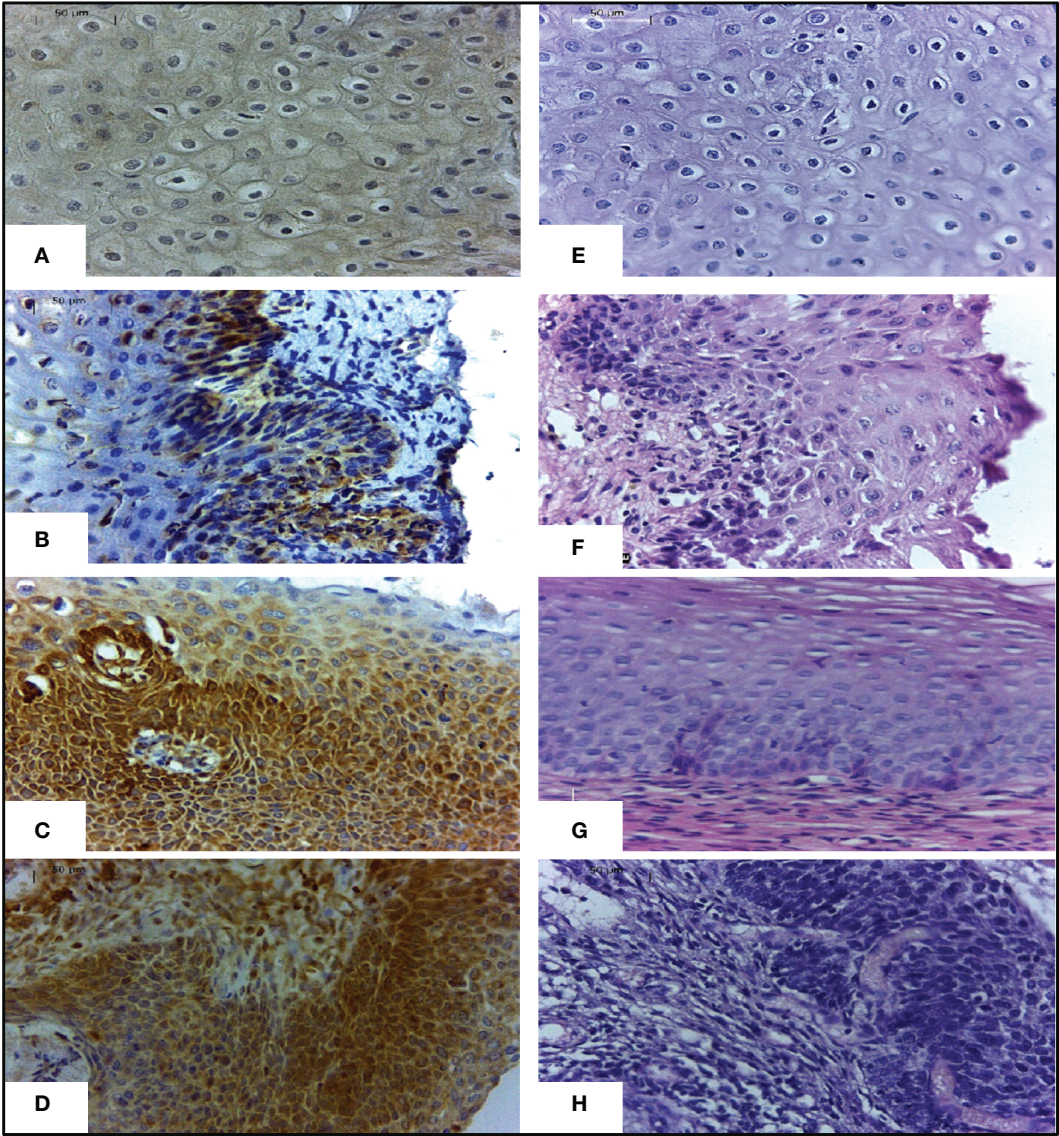
Expression of TAP-2 protein in cervical tissues stratified according to the severity of the lesion.Legend: Immunohistochemical staining with anti-TAP-2 (left images) in cervical biopsy specimens with a benign lesion **(A)**, CIN 1 **(B)**, CIN 2 **(C)**, and CIN 3 **(D)**. Labeling intensity progressively increases from mild to strong according to the severity of the cervical lesion. H&E staining (right images) cervical biopsy specimens with a benign lesion **(E)**, CIN 1 **(F)**, CIN 2 **(G)**, and CIN 3 **(H)**. Magnification ×400.

The TAP-2 levels increased according to the severity of the cervical lesion and were significantly higher in tissues with CIN 3 compared to CIN 1 (P=0.0483) or BL (P=0.0001) (**Figure 2A**). It was not clear the difference in TAP-2 levels according to the cellular ploidy (P=0.0521) (**Figure 2B**), but the levels a significantly high in HPV+ samples (P<0.0001) (**Figure 2C**). The TAP-2 protein exhibited a focal and diffuse cytoplasm expression in tissue lesions (**Figure 3**).

**Figure 4:**
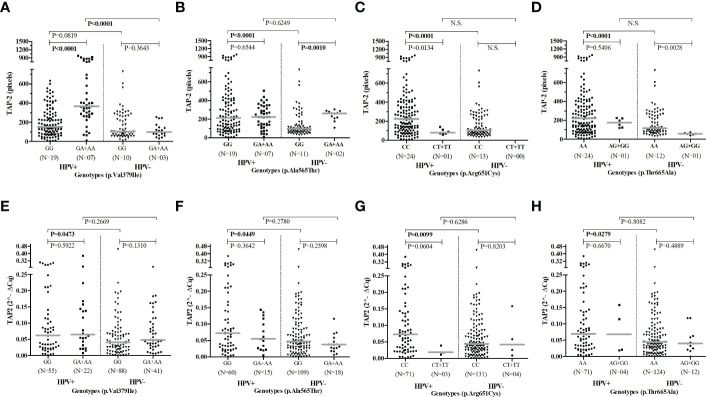
Evaluation of *TAP2* genotypes according to gene and protein expression stratified by HPV infection.Legend: Number of patients analyzed (N). Presence of HPV infection (HPV+). Absence of HPV infection (HPV-). Note: The Mann-Whitney test was used to estimate the differences between the expressions of TAP-2 protein in cervical biopsy samples **(A–D)** and *TAP2* mRNA in cervical exfoliative samples **(E–H)**.

Regarding the HPV infection, there was no significant difference in the allelic and genotypic frequencies of the *TAP2* p.Val379Ile, p.Ala565Thr, p.Arg651Cys, and p.Thr665Ala SNPs in HPV+ compared to HPV- women. The HPV infection increased the *TAP2* mRNA expression in exfoliative cervical cells and the levels of TAP-2 protein in the cervical lesion of carriers of the common homozygous p.Val379Ile GG, p.Ala565Thr GG, p.Arg651Cys CC, and p.Thr665Ala AA genotypes. However, women carrying rare genotypes responded differently to the HPV infection. Carriers of the rare p.Val379Ile A allele showed a significant increase in tissue TAP-2 protein, while carriers of the p.Ala565Thr A allele showed no changes in TAP-2 levels. Additionally, this genotype was associated with higher TAP-2 protein levels than that observed in carriers of the common genotype (p.Ala565Thr GG) without HPV infection (**Figure 4**).

### 3.3 The risk of the *TAP2* gene haplotypes and diplotypes on the development of the cervical lesion

**Figure 5:**
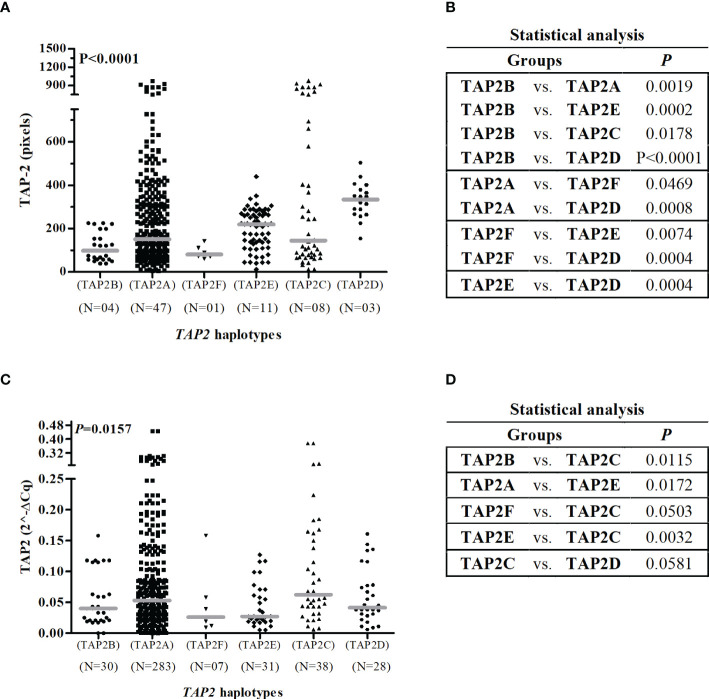
Gene and protein expression of TAP2 stratified by *TAP2* gene haplotypes.Legend: Number of haplotypes observed (N). Note: The Mann-Whitney test was used to estimate the differences between the expressions of TAP-2 protein in cervical biopsy samples **(A, B)** and *TAP2* mRNA in cervical exfoliative samples **(C, D)**.

This analysis excluded 13 of the 296 participants due to the lack of one SNP genotyping, totaling 283 women exhibiting seven haplotypes. No haplotype was associated with susceptibility to HPV infection. The most frequent haplotype was the TAP2A haplotype (68.6%), formed by the combination of wild-type alleles, followed by the TAP2C haplotype (8.5%) (**Table 4**). The TAP2A haplotype was associated with protection against the development of aneuploidy (OR=0.4; 95%Cl=0.2-0.9; P=0.0199) and with protection against the development of HSIL (OR=0.4; 95% CI=0.3-0.8; P=0.0021). The TAP2A and TAP2C haplotypes diverge only at the position of the Val379Ile SNP (**Table 4**). Considering that these haplotypes were the most frequent ones and exhibited closely similar TAP-2 protein levels and *TAP2* gene expression, we assumed the levels produced by these haplotypes as standards (**Figure 5**).

**Table 4:** The *TAP2* haplotypes and diplotypes associated with cell ploidy and cytological and histological alterations.

*TAP2* gene haplotypes	Cell ploidy								Cytological alterations						Histological alterations					
	^a^Patients (N=283)		Aneuploidy (N=21)		Diploidy (N=93)		*P*	*OR (CI-95%)*	HSIL (N=73)		No atypias (N=92)		P	*OR (CI-95%)*	CIN (N=154)		Benign lesion (N=37)		*P*	*OR (CI-95%)*
	n = 566	(%)	n = 42	(%)	n = 186	(%)			n = 146	(%)	n = 184	(%)			n = 308	(%)	n = 74	(%)		
**GGCA (TAP2A)**	388	68.6	21	**50**	129	**69.4**	**0.0199**	**0.4 (0.2-0.9)**	86	**58.9**	138	**75**	**0.0021**	**0.4 (0.3-0.8)**	205	66.6	52	70.3	0.5833	0.8 (0.5-1.4)
**GGCG (TAP2B)**	42	7.4	2	4.8	19	10.2	0.3812	0.4 (0.09-1.9)	20	13.7	15	8.2	0.1094	1.7 (0.8-3.6)	30	9.7	4	5.4	0.3616	1.8 (0.6-5.5)
**AGCA (TAP2C)**	48	8.5	6	14.2	14	7.5	0.2214	2.0 (0.7-5.7)	10	6.8	11	6	0.822	1.1 (0.5-2.8)	28	9.1	5	6.8	0.6483	1.3 (0.5-3.7)
**AACA (TAP2D)**	29	5.1	7	**16.7**	4	**2.2**	**0.0008**	**9.1 (2.5-32.7)**	7	4.8	11	6	0.8081	0.8 (0.3-2.0)	10	**3.2**	9	**12.2**	**0.0043**	**0.2 (0.1-0.6)**
**GACA (TAP2E)**	44	7.8	5	11.9	15	8.1	0.3815	1.5 (0.5-4.5)	18	**12.3**	6	**3.3**	**0.0023**	**4.1 (1.6-10.8)**	29	**9.4**	1	**1.4**	**0.0158**	**7.6 (1.0-56.6)**
**GGTA (TAP2F)**	14	2.5	1	2.4	5	2.7	1.000	0.8 (0.1-7.7)	5	3.4	3	1.6	0.4739	2.1 (0.5-9.1)	5	1.6	3	4.1	0.1876	0.4 (0.09-1.6)
**AGCG (TAP2G)**	1	0.2	0	0	0	0	NA		0	0	0	0	NA		1	0.3	0	0	NA	
***TAP2* gene diplotypes**	^a^Patients		**Aneuploidy**		**Diploidy**		***P***	***OR (CI-95%)***	**ASC-US, ASC-H, and LSIL**		**HSIL**		***P***	***OR (CI-95%)***	**CIN**		**Benign lesion**		***P***	***OR (CI-95%)***
	**N = 228**	**(%)**	**N= 22**	**(%)**	**N= 92**	**(%)**			**N=73**	**(%)**	**N = 73**	**(%)**			**N= 153**	**(%)**	**N = 37**	**(%)**		
**GGCG/GGCG (TAP2B/TAP2B)**	20	7.1	1	4.5	9	9.8	0.6841	0.4 (0.05-3.6)	2	**2.7**	10	**13.7**	**0.0311**	**5.6 (1.2-26.7)**	14	9.2	2	5.4	0.7417	1.7 (0.4-8.1)
**GGCA/GGCA (TAP2A/TAP2A)**	154	54.4	7	**31.8**	52	**56.5**	**0.0562**	**0.3 (0.1-0.9)**	41	56.2	33	45.2	0.2465	1.5 (0.8-2.9)	82	53.6	20	54.1	1.000	0.9 (0.5-2.0)
**GGCA/AACA (TAP2A/TAP2D)**	17	6.0	3	13.6	3	3.3	0.0849	4.4 (0.8-25.0)	4	5.5	4	5.5	1.000	1.0 (0.2-4.1)	4	**2.6**	7	**18.9**	**0.0012**	**0.1 (0.03-0.4)**
**GGCA/AGCA (TAP2A/TAP2C)**	37	13.1	2	9.1	12	13.0	1.000	0.6 (0.1-3.2)	13	17.8	7	9.6	0.2282	2.0 (0.7-5.4)	21	13.7	3	8.1	0.5805	1.8 (0.5-6.4)
**Other diplotypes**	55	19.4	9	41.0	16	17.4	NA		13	17.8	19	26.0	NA		32	20.9	5	13.5	NA	

Haplotypes were constructed with the TAP2 SNPs rs1800454-rs2228396-rs4148876-rs241447. For example, haplotypes G-G-C-A represents G allele of rs1800454, the G allele of rs2228396, the C allele of rs4148876, and the A allele of rs241447. Note: in parentheses, the haplotype nomenclature by Ivansson et al. (2008) (Ben-David and Amon, 2020). CI, Confidence Interval; OR, Odds Ratio.

a Number of patients studied, n = number of chromosomes evaluated. Number of individuals (N). NA, means not applicable. The results highlighted in bold are statistically significant. Fisher’s exact test calculated the P-value, and P<0.05 was considered statistically significant.

The TAP2E and TAP2D haplotypes diverge only in the position of the p.Val379Ile SNP, with no difference in mRNA expression. However, they are great protein producers, and the TAP2D showed the highest protein level (P=0.0004) (**Figure 5**). The TAP2D haplotype was associated with a risk for aneuploidy (OR=9.1; 95%Cl=2.5-32.7; P=0.0008), and the TAP2E haplotype was associated with the risk of HSIL (OR=4.1; 95% CI=1.6-10.8; P=0.0023) (**Table 4**). In addition, the TAP2E haplotype was associated with risk (OR=7.6; 95% CI=1.0-56.6; P=0.0158), and TAP2D with protection against the development of CIN (OR=0.2; 95% CI=0.1-0.6; P=0.0043), which may indicate an imbalance of cases with lesions of different severity within each group, impacted by the reduced number of biopsy samples.

The TAP2B haplotype was associated with low *TAP2* mRNA and protein levels. The rare p.Thr665Ala allele G is the unique difference observed about the TAP2A haplotype sequence, suggesting its role in lowering the *TAP2* expression.

To investigate the effect of haplotype combinations, 14 diplotypes were identified for the *TAP2* gene; ten were considered rare because of their low frequencies. The TAP2A/TAP2A diplotype (54.4%) was associated with protection against aneuploidy (OR=0.36; 95% CI=0.13-0.96), while TAP2B/TAP2B diplotype (7%) was associated with a 5.6-fold increase in the risk of HSIL (P=0.0311; 95% CI=1.2-26.7). The TAP2A/TAP2D diplotype (6%) was associated with protection against CIN (OR=0.11; CI=0.03-0.41; P=0.0012), suggesting the effect of the TAP2A haplotype was dominant (**Table 4**).

**Figure 6:**
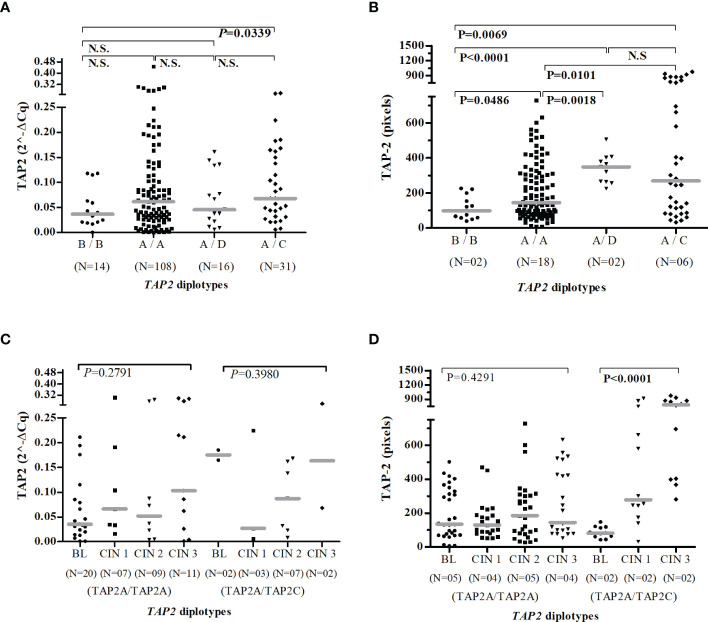
Gene and protein expression of TAP2 stratified according to the *TAP2* gene diplotypes. Number of patients evaluated (N). TAP2B/TAP2B (B/B); TAP2A/TAP2A (A/A); TAP2A/TAP2D (A/D); TAP2A/TAP2C (A/C). Evaluation of *TAP2* expression in mRNA **(A, C)** and protein **(B, D)**. Presence of HPV infection (HPV+). Absence of HPV infection (HPV-). The Mann-Whitney or Kruskal-Wallis tests were used to estimate the differences between the TAP-2 expressions in the evaluated groups.

Considering the TAP-2 protein levels produced by carriers of TAP2A/TAP2A diplotype as standard, women carriers of the TAP2B/TAP2B (P=0.0486) showed the lowest protein level, and those who encompassed the TAP2A haplotype combined to TAP2D (P=0.0018) or TAP2C (P=0.0101) haplotypes showed increased protein levels (**Figure 6B**). In addition, the increased TAP-2 protein in carriers of the TAP2A/TAP2C diplotype showed an apparent relationship between TAP-2 level and lesion severity (CIN 3) (**Figure 6D**).

Moreover, HPV infected carries of the TAP2A/TAP2A diplotype expressed more *TAP2* mRNA transcripts than that HPV- (P=0.0155), and a higher median of TAP-2 protein levels was found in HPV+ women with TAP2A/TAP2C diplotype when compared to HPV- women (P<0.0001) (**Figure S2**).

## 4 Discussion

The recognition, processing, and presentation of antigens to immune cells are complex and require a well-orchestrated activity of several genes to eliminate the aggressor because infectious agents have developed mechanisms to evade the immune response, adapting to the adversities of the environment and persisting in their parasitism (Estêvão et al., 2019; Mantel et al., 2022). In this study, we showed that TAP tissue levels changed in HPV infection and that *TAP* gene variants modulated *TAP* expression, which may contribute to the progression of cervical lesions.

The T*AP1* and *TAP2* genes are conserved with few mutations in the coding region assigned in the HLA database, with six and five isoforms identified, respectively (Robinson et al., 2020). As most of the reported mutations are in the cytosolic nucleotide-binding domains (NBDs) of the TAP heterodimeric complex, in addition to being possible changes in mRNA expression and structural stability and at the protein level, variants of the *TAP* gene can cause functional alterations, affecting ATP binding capacity and peptide transport efficiency (Lapinski et al., 2001; Lankat-Buttgereit and Tampé, 2002).

In the *TAP1* gene, the p.Ile333Val SNP in exon-4 and the p.Asp697Gly SNP in exon-10 are the most studied due to their association with disease. The p.Ile333Val A allele was not reported in other primates, suggesting associated with human evolution, and is in LD with the Asp697Gly A allele (Tang et al., 2001). Because the p.Asp697Gly variant was mapped to the NBD region and was associated with recurrent respiratory papillomatosis in Mexicans (Palomares-Marin et al., 2020) and with cervical lesions in Americans and Europeans (Mehta et al., 2007; Deshpande et al., 2008), we focused on studying it. We showed that the frequency of the G allele for the p.Asp697Gly SNP of *TAP1* in northeastern Brazilians (18%) was similar among Europeans (14.4%), Africans (23.3%), Asians (16.5%), Latin Americans with Afro-Caribbean ancestry (16.3%), and Latin Americans with European and Native American ancestry (17.6%) (Phan et al., 2020).

We demonstrated that women who carry the G allele in homozygosis (3%) have a higher risk of having aneuploidy in cervical cells (**Table 2**). In the acute phase of HPV infection, the viral genome remains in episomal form within the infected epithelial cell; during epithelial desquamation, recognizing the HPV antigen increases the inflammatory infiltrate. Integration of the HPV genome into the human genome leads to genetic instability and aneuploidy of cervical cells (Martins et al., 2014; Li et al., 2018). Integration is also responsible for activating viral oncoproteins, which co-opt effector molecules of the immune system, contributing to the progression to malignancy (Estêvão et al., 2019).

Our findings also showed a relationship between aneuploidy and low levels of TAP-1 (**Figure 1C**). Studies have explicitly shown the downregulation of *TAP1* and *HLA-I* expression by HPV-16 E7 and HPV-18 E7 oncoproteins (Georgopoulos et al., 2000; Vermeulen et al., 2007; Li et al., 2010). HPV-16 E5 also interacts with the heavy chain of the HLA-I molecule through the hydrophobic region of the E5 protein and retains HLA-I in the Golgi and ER (Ashrafi et al., 2006). The lower expression of TAP, resulting from the inhibition of gene transcription, protein translation, or post-translational events, decreases the presentation of viral peptides coupled to HLA-I molecules on the cell surface, decreasing the activation of cytotoxic T cells and inflammation, consequently, viral elimination. In this case, HLA-I molecules can present peptides from the membrane, cytosolic, or secreted proteins through a less efficient TAP-independent mechanism, which may also contribute to the virus evading the NK cell-mediated immune response (Mantel et al., 2022).

Unfortunately, we cannot draw strong conclusions from the results with TAP-1 because the analysis at the protein level was compromised by the small number of samples analyzed. Also, there was no significant difference associating the mRNA expression level with cervical lesions, aneuploidy, or the presence of HPV infection. As a preliminary analysis of the data, we could hypothesize that the presence of HPV increases tissue TAP-1 expression (**Figure 1D**), which is in line with the increase in protein in the inflammatory phase of the low-grade lesion (**Figure 1A**) in response to the antigenic stimulus (Gostout et al., 2003; Kelly and Trowsdale, 2019). Still, we cannot confirm the association of the mutant allele with the reduction in protein levels since the association between the allele and aneuploidy may not be associated with expression intensity but with transport efficiency; this was not assessed in this study (Praest et al., 2018). The mutant G allele encodes an aspartic acid residue at position 697 of the TAP-1 protein, close to the peptide binding site, which may influence the peptide-TAP-1 interaction and its transport to the endoplasmic reticulum (Quadri and Singal, 1998).

In this study, we also evaluated the association of *TAP2* variants with aneuploidy and the severity of the cervical lesions, irrespective of the *TAP1* gene. The Val379Ile SNP codes an amino acid localized in the transmembrane domain region (TMD2) of the TAP-2 protein, and the p.Ala565Thr, p.Arg651Cys, and p.Thr665Ala SNPs code for amino acids in the cytosolic nucleotide-binding domain (NBD2) of TAP-2 (Praest et al., 2018). We showed that the rare A allele for *TAP2* p.Val379Ile and p.Ala565Thr SNPs, in single or double doses, were associated with the risk of cervical cell aneuploidy, considered a hallmark of cervical lesion progression (Ben-David and Amon, 2020). In addition, Brazilian carriers of the rare G allele for p.Thr665Ala SNP were at risk for developing CIN and HSIL.

Regarding the presence of HPV, carriers of the wild-type genotype for all SNPs studied in homozygosis showed increased expression of mRNA and TAP-2 protein as part of the elaboration of the immune response to the infection. However, carriers of the p.Ala565Thr mutant allele are high producers of TAP-2, suggesting that the protein is more stable, as mRNA and protein levels were not affected by the viral infection (**Figure 4B**). On the other hand, carriers of the mutant p.Val379Ile allele respond to the presence of HPV with a more expressive increase in protein levels than those produced by carriers of the wild-type allele in response to the presence of HPV (**Figure 4A**), suggesting a post-translational modulation in the expression of *TAP*, since the mRNA levels were not altered by the HPV infection (Pedroza-Torres et al., 2014; Lazaridou et al., 2020).

Controversies about the association of *TAP1* and *TAP2* gene variants with the risk of cervical cancer may arise from the ethnic diversity of the studied populations, whether the study was in precancerous or cancerous lesions, and depending on the parameter used for the comparison between groups, that is, cytological or histological changes, or abnormal cervical cell ploidy. For example, in Austrian women, the *TAP1* p.Asp697Gly SNP showed no association with CIN compared to healthy controls (Natter et al., 2013). However, the G allele was associated with a reduced risk of HSIL (CIN 2 and CIN 3) in American women infected by HPV (Einstein et al., 2009); and protection against the development of cervical cancer in Chinese women (Yang et al., 2021). Another example is the lack of association between the SNPs TAP2, p.Val379Ile, p.Ala565Thr, and p.Arg651Cys, with histological and cytological alterations and cervical cancer reported in Chinese (Yang et al., 2021) and Swedish (Ivansson et al., 2008) women when compared to the control group. However, the TAP2 SNP p.Val379Ile A allele was associated with the risk of esophageal cancer in Chinese patients with HPV+ (Cao et al., 2005) and with cervical cell aneuploidy in our study population. Also, the T allele of the SNP TAP2 p.Arg651Cys was associated with an increased risk of cervical cancer in women from Bali, a region of Indonesia (Mehta et al., 2015), while in Dutch women with HPV+, the risk was attributed to the C allele (Mehta et al., 2007).

Regarding the p.Thr665Ala SNP of the *TAP2* gene, the women’s ancestry also influenced cancer susceptibility, especially cervical cancer (Liu et al., 2019). We found that Brazilian carriers of the rare G allele were at risk for developing CIN and HSIL. In contrast, the A allele of the p.Thr665Ala SNP was associated with cervical cancer in Swedish women (Ivansson et al., 2008). In addition, neither allele was related to cervical cancer in New Yorkers (Einstein et al., 2009) and Austrian (Natter et al., 2013) women. A study of 1,306 Swedish women with cervical cancer showed a strong linkage disequilibrium between the *TAP2* p.Thr665Ala and *DQB1* alleles. The authors investigated the distribution of *TAP2* alleles among women that expressed DQB1 risk (*0301, *0402, *0602) or protection alleles (*0501 and *0603), showing less difference in *TAP2* alleles distribution between cases and controls among carriers of the *DQB1* risk alleles, suggesting a hitchhiking effect of *TAP2* with *DQB1* alleles (Ivansson et al., 2008). This may explain the controversies in the association of the *TAP2* p.Thr665Ala allele with the risk of cervical cancer in different populations. Moreover, the HLA system comprises a large region with many genes in linkage disequilibrium. Therefore, other genes in different populations may modulate the association between the presence of a specific allele and the severity of the cervical lesion (Dawkins et al., 1999).

Since the effect of each allele may differ, and the resultant product is more important for evaluating the impact on the evolution of the cervical lesion (Mehta et al., 2007), we considered the sequence of each haplotype observed in our population to assess its clinical significance (**Table 5**). The lower genetic diversity may result from selecting more efficient variants for the best innate or adaptive immune response against environmental pathogens (Zeberg and Pääbo,). This is the case of the TAP2A haplotype formed by the wild-type homozygous genotypes of the polymorphic sites of the regions responsible for peptide binding and transport across the endoplasmic reticulum membrane, which corresponds to 68.6% of cases. Considering that the TAP2A and TAP2C haplotypes account for 77.1% of the identified types and because the median TAP-2 levels in tissue were similar, we assumed these levels as standard and classified them as intermediate. The TAP2A and TAP2C haplotypes differ only in the p.Val379Ile SNP. The TAP-2 levels associated with the TAP2D and TAP2E haplotypes, which correspond to 12.9% of the observed haplotypes, were higher, and carriers were at risk for HSIL and aneuploidy. The sequences of the TAP2D and TAP2E haplotypes also differ only in the p.Val379Ile SNP, suggesting that this SNP may not be directly related to the increase in TAP-2. The minor allele A of the p.Ala565Thr SNP seems to play a role in the increase of TAP-2 levels in TAP2D and TAP2E carriers, while the mutant allele G of the p.Thr665Ala SNP is involved in the decrease of the protein in TAP2B carriers, whose are also at increased risk of serious injury. Few association studies on TAP2 haplotypes and cervical cancer are available. In one of them, Natter et al. (2013) also reported that Caucasian women from Austria with TAP2C haplotype were at lower risk for the development of CIN (Natter et al., 2013).

**Table 5:** *TAP2* haplotypes and their clinical impact.

Haplotype							Median protein levels	Effect
Name	n = 566	Frequency	Sequence					
*TAP2A*	388	68.6%	G	G	C	A	Intermediate	Protection
*TAP2C*	48	8.5%	A	G	C	A	Intermediate	Protection
*TAP2D*	29	5.1%	A	A	C	A	High	Risk aneuploidy
*TAP2E*	44	7.8%	G	A	C	A	High	Risk HSIL
*TAP2B*	42	7.4%	G	G	C	G	Low	Risk HSIL
*Other haplotypes*	15	2,6%	-	-	-	-	-	-

The TAP2A/TAP2A carriers responded to HPV infection, increasing the *TAP2* mRNA transcripts compared to women carriers non-infected but without a corresponding protein level, which may indicate an increase in the protein turnover or a viral posttranslational regulation. Gameiro et al. (2017) analyzed *TAP1* and *TAP2* expression using TCGA (The Cancer Genome Atlas) data and found higher expression in cervical carcinoma tumors in HPV+ samples than in normal control tissue (Gameiro et al., 2017). *TAP1* and *TAP2* expressions were also higher in EBV-associated gastric carcinoma samples than in normal control tissues or other gastric carcinoma subtypes (Ghasemi et al., 2020). Interestingly, the TAP2A/TAP2C carriers responded to the HPV infection with unchanged *TAP2* expression and a concomitant increase in protein levels. The rare allele A of the p.Val379Ile SNP in the TAP2C haplotype elevates the TAP-2 level in HPV-infected tissue and CIN 3 samples.

The increase in TAP2 expression and protein level may be related to greater recognition and processing of the viral peptide, increasing the adaptive immune response (Thomas and Tampé, 2021). Nevertheless, the viral modulation of *TAP2* expression, possibly mediated by p.Val379Ile SNP allele A, may contribute to cervical cancer progression from precursor lesions (Fowler and Frazer, 2004; Mpakali and Stratikos, 2021).

Some viruses have an immune response evasion mechanism based on the release of inhibitory molecules, which decrease TAP levels (Estêvão et al., 2019; Mantel et al., 2022). In the reduction of TAP, the viral peptide is less efficiently transported into the endoplasmic reticulum, and the assembly of the HLA-peptide complex is decreased. The empty HLA-I molecule is unstable and degraded, leading to its depletion at the cell surface for antigen presentation to cytotoxic T cells. However, it was observed that some HLA-B allotypes had increased expression in the absence or decrease of TAP. These allotypes are stable and highly efficient in peptide loading for a broad specificity of peptides, allowing to augment 5 to 10-fold the viral peptide presentation on the surface through a mechanism independent of TAP (Geng et al., 2018).

The main limitation of our study was the low number of biopsies, which compromised the TAP-1 and TAP-2 protein analyses, reducing the possibility of multiple comparisons. Nevertheless, the evaluation of mRNA expression in exfoliative cervical cell samples allowed the analysis of a more significant number of samples, increasing the strength of the observed association. In addition, we chose to use the DNA index in the final association analysis because aneuploidy is related to the integration of the HPV genome into cervical cells, and the increase in cervical dysplasia has a prognostic value for cervical cancer (Heselmeyer et al., 1996; Ben-David and Amon, 2020).

## 5 Conclusion

The results obtained with the diplotypes formed by the four SNPs in the *TAP2* gene allowed the identification of low, intermediate, and high TAP-2 producers in women. We found the association of some haplotypes with aneuploidy and the presence of HSIL. We showed the increase of TAP-2 protein in HPV+ and CIN 3 cervical lesions, a physiologic mechanism for eliminating infected and damaged cells. In addition, we addressed the possible mechanism of immune evasion exerted by HPV in modulating the expression of *TAP* variants, which may favor viral persistence, contributing to the evolution of the disease to malignancy. Further functional studies will confirm the relationship between *TAP* gene variants and *TAP2* expression, HPV infection, and disease outcome.

## Data availability statement

The data presented in the study are deposited in the Figshare repository, and accession links and DOIs can be found below: https://doi.org/10.6084/m9.figshare.21263343, https://doi.org/10.6084/m9.figshare.21265785, https://doi.org/10.6084/m9.figshare.21265848.v1.

## Ethics statement

The studies involving human participants were reviewed and approved by The Ethics Committee of the Instituto Aggeu Magalhães approved this study under the protocol CAAE:51111115.9.0000.5190. The patients/participants provided their written informed consent to participate in this study.

## Author contributions

FM, NL-S conceived, designed the study, did the formal analysis, and wrote the paper. FM, MS, NS, TG, RG, FG, LP conducted the experimental work. FM, LP, MR, SW, MM followed-up patients and performed cytopathological and coloscopy evaluations. NL-S and ED applied for financial support and managed the project. All authors contributed to the article and approved the submitted version.
