# Supplementary material for: The antigen processing-associated transporter gene polymorphism: Role on gene and protein expression in HPV-infected pre-cancerous cervical lesion
Source: Front Cell Infect Microbiol. 2022 Dec 21;12:979800. doi: 10.3389/fcimb.2022.979800 (PMC9811671; doi:10.3389/fcimb.2022.979800)
Supplement: Supplementary file 1 [file Table_1.docx]

Supplementary Material

# Supplementary Figures

# Figure S1 - Analysis of *TAP2* gene expression according to the genotypes of the four SNPs evaluated.

| A)   | B) |
| --- | --- |
| C)   | D)   |

**Supplementary Figure 1.** Legend: Number of patients evaluated (N). The Mann-Whitney or Kruskal-Wallis tests were used to estimate the differences between the *TAP2* mRNA expressions in the evaluated groups.

**Figure S2 -** Gene and protein expression of *TAP2* gene diplotypes according to HPV infection and cell ploidy

| A)   |
| --- |
| B)   |

**Supplementary Figure 2.** Legend: Number of patients evaluated (N). Presence of HPV infection (HPV+). Absence of HPV infection (HPV-). Evaluation of *TAP2* expression in mRNA (A) and protein (B). The Mann-Whitney or Kruskal-Wallis tests were used to estimate the differences between the TAP-2 expressions in the evaluated groups.
